# The Practical Potential of Bacilli and Their Enzymes for Industrial Production

**DOI:** 10.3389/fmicb.2020.01782

**Published:** 2020-08-04

**Authors:** Iuliia Danilova, Margarita Sharipova

**Affiliations:** Department of Microbiology, Institute of Fundamental Medicine and Biology, Kazan Federal University, Kazan, Russia

**Keywords:** microbial enzymes, proteases, *Bacillus*, applications, medicine, agriculture, microbiology

## Abstract

*Bacillus* spp. are an affordable source of enzymes due to their wide distribution, safety in work, ease of cultivation, and susceptibility to genetic transformations. Researchers are particularly interested in proteolytic enzymes, which constitute one of the most diverse groups of microbial proteins in terms of properties. Despite the long history of their research, this group of enzymes continue to show great potential for practical application in the biomedical industry, as well as in the agricultural industry. Thus, the unique properties of bacillary proteinases, such as stability in a wide range of temperatures and pH, high specificity, biodegradability of a wide range of substrates, and the high potential of sequenced *Bacillus* genomes are a powerful foundation for the development of new biotechnologies. The current review aims to discuss recent studies on various enzymes in particular, proteinases produced by bacteria of the genus *Bacillus*, along with their prospective practical applications. This article also presents an interpretive summary of the recent developments on the usage of probiotic *Bacillus* strains as potential feed additives.

## Introduction

Enzyme-based technologies guarantee efficient utilization of raw materials and generation of minimal or no wastes and shun the usage of toxic chemicals (Singh and Bajaj, 2016, 2017). The industrial application of enzymes may contribute significantly toward the development of environmentally benign processes (Mhamdi et al., 2017). The main advantages of enzymatic methods include: reduced energy requirements, increased product yield, increased catalytic efficiency, and reduced catalyst losses and by-products. Peptide hydrolases or proteases refer to a large group of proteolytic enzymes that hydrolyze peptide bonds in proteins and peptides (Gurumallesh et al., 2019). The scope of proteinase research is steadily increasing due to the abundance of information on the microbial genome that paves way for new techniques based on genetic engineering, genomics and proteomics. Bacteria are the most dominant group of protease producers with the genus *Bacillus* being the most prominent source (Mienda et al., 2014). This is mainly due to the high capacity for protein secretion that several *Bacillus* species possess (Harwood and Cranenburgh, 2008; Cui et al., 2018). In addition, different species of the *Bacillus* genus produce neutral and alkaline proteases (Anandharaj et al., 2016; Rehman et al., 2017), which is interesting for enzyme use in industry. Another approach is genetic engineering of proteases from *Bacillus* sp., strains, resulting in proteases with differentiated properties. Additionally, *Bacillus* species have been studied for the heterologous production of proteases from other microorganisms, including different *Bacillus* species (Cui et al., 2018). *Bacillus* proteases have several remarkable characteristics for many industrial applications such as broad pH and temperature activity, stability range, alkaline and toxic compounds tolerance, including oxidants and surfactants (Contesini et al., 2018). Based on these interesting aspects, proteases from the genus *Bacillus* are used in the production of detergents, food products, leather, silk, and agrochemical products (Dos Santos Aguilar and Sato, 2018; Razzaq et al., 2019). The alkaline serine protease (DHAP) from *Bacillus pumilus* BA06 is a typical mesophilic enzyme, which has demonstrated great potential in various industrial applications (Zhao and Feng, 2018). The multiple applications presently using alkaline proteases are reported in the article of Kamal et al. (2016). It focuses on the updated tidings on nutritional effects, physiochemical parameters, biochemical aspects, and strain improvement methodologies for hyperproduction of protease (Kamal et al., 2016). In the review of Gurumallesh et al. (2019), the potential utilizations of the enzyme in a variety of industries is described.

Probiotics use in agriculture has gained attention as microbial candidates to maintain the health and the wellbeing of many animals. Among the many microbial candidates, probiotic *Bacillus* has sporulation capacity that makes them survive harsh environmental conditions, is non-pathogenic and non-toxic when fed to animal, and can produce antimicrobial substances making them more suitable candidates compared to other probiotics (Kuebutornye et al., 2019).

*Bacilli* and their proteases have received renewed attention due to increasing demands in medical and industrial commodities. Not so much data on *Bacillus* proteinase-based drugs with prospective applications in agriculture and medicine can be found in the literature. For instance, Razzaq et al. (2019) confirmed the potential use of proteinase-based preparations in industry, for the production of textiles, leather, food, and detergents.

This manuscript summarizes data on *Bacillus* enzymes, which have potential applications in agriculture and pharmaceutical industries, especially in thrombolytic therapy and the treatment of neurodegenerative diseases.

## The Use of Microbial Enzymes in Medicine

The lack of therapeutic agents in areas such as cardiovascular medicine and the treatment of neurodegenerative diseases remains the basis in the search for novel therapeutic (enzymatic) drugs. Microbial enzymes are used in the production of beta-lactam antibiotics, such as semisynthetic penicillins and cephalosporins (Volpato et al., 2010). Some drugs used to lower blood cholesterol are likewise based on microbial enzymes (Ma et al., 2010).

Microbial enzymes can be directly used as medications. In therapy, lysing agents, such as streptokinases (*Streptococcus haemolyticus*) and staphylokinases (some strains of *Staphylococcus aureus*; Jespers et al., 1999), are widely used as fibrinolytics in treating thrombosis. Terrilitins (*Aspergillus tericola*) are known to aid in the treatment of wounds, trophic wounds, and burns (Shataeva et al., 1973; Khmelevskaia, 2000). Additionally, lipases (*Bacillus*, *Acinetobacter*, *Propionibacterium*, *Chromobacterium*, and *Alkaligenes*) hydrolyze fats in chronic gastrointestinal diseases (Hasan et al., 2006). Alkaline proteases from *Bacillus subtilis* act as efficient transdermal drug delivery system for dermatological applications (Nounou et al., 2017). Furthermore, amylases (*Bacillus*) hydrolyzes starch used for pancreatic insufficiency, etc., (Souza and Magalhaes, 2010; Jujjavarapu and Dhagat, 2019).

At present, most biological products are obtained based on genetically modified organisms. The main producers of recombinant enzymes are bacteria, such as *Escherichia coli*, *B. subtilis*, *Lactobacillus lactis*, and yeast, including *Pichia pastoris* (Baneyx, 1999; Yang et al., 2011; Garcia-Fruitos, 2012; Suleimanova et al., 2016; Cui et al., 2018; Troshagina et al., 2018). Сardiovascular and oncological diseases, as well as Alzheimer’s disease (AD), are among the most common causes of death. The treatment of these formidable diseases continues to be one of the most pressing issues in modern medicine. This necessitates the search for new potential microbial proteins for prospective use in medicine and pharmacy.

### Fibrinolytic Enzymes of the Genus *Bacillus*

Fibrinolytic proteases are a promising alternative to existing drugs for thrombolytic therapy. Fibrinolytic enzymes are obtained from diverse microorganisms, the most important of which are representatives of the genus *Bacillus*. Less than two decades ago, the enzyme preparation thrombovazim was developed by Plotnikov et al. (2009) in Russia that based on the subtilase of *B. subtilis*, which has since then been adopted for the treatment of acute myocardial infarction and unstable angina (Plotnikov et al., 2009). Thrombovazim is a *B. subtilis* proteinase preparation immobilized on polyethylene oxide and has no analoges among the drugs with similar mechanism of action. Thrombovazim has low toxicity, which grants the possibility of its use in both hospital and extramural settings (Plotnikov et al., 2009).

However, the lack of efficient thrombolytics is a primary factor, which drives the intensive hunt for new enzymes from microorganisms with high thrombolytic activity. Subtilisin DFE, a serine proteinase with fibrinolytic properties, is expressed in the protease-deficient strain of *B. subtilis* WB600 (Peng et al., 2004). Proteinases QK-1 and QK-2 obtained from *B. subtilis* QK02 and a subtilisin-like proteinase acquired from *B. subtilis* TP-6 have a high level of fibrinolytic activity and are promising for thrombolytic therapy (Ko et al., 2004; Kim et al., 2006). For instance, in the subtilisin-like proteinase FS33 isolated from *B. subtilis* DC33, the level of fibrinolytic activity was six times higher than the activity level of Carlsberg subtilisin. This enzyme destroyed fibrin clots by forming active plasmin from plasminogen, as well as by direct lysis of fibrin clots (Wang et al., 2006). Several serine fibrinolytic proteinases have been described such as: proteinases secreted by *B. subtilis* LD-8547 (Wang et al., 2008); subtilisin BSF1 from *B. subtilis* A26 and BAF1 produced by *Bacillus amyloliquefaciens* An6 (Agrebi et al., 2009, 2010); recombinant subtilisin of *B. subtilis* PTCC 1023 expressed in *E. coli* BL21 (DE3; Ghasemi et al., 2012); and subtilisin-like serine proteinase isolated from the marine strain *B. subtilis* ICTF-1 (Mahajan et al., 2012); as well as the enzyme secreted by *Bacillus cereus* SRM-001 (Narasimhan et al., 2018). Serine proteinases of *B. pumilus* 7P have fibrinolytic activity but are unable to activate plasminogen. Beyond that, the thrombolytic activity of these enzymes has been shown to be dose-dependent (Danilova et al., 2012). Currently, the *B. subtilis* C10 strain is considered prospective for cost-effective production of fibrinolytic serine protease (Thu et al., 2019). The strain *Bacillus velezensis* BS2, which was isolated from marine squirt (munggae) jeotgal, produces an enzyme that exhibits strong fibrinolytic activity (Yao et al., 2019).

In the event of an abridged function of the body’s anticoagulant system, the lysis of one blood clot does not rule out the formation of new blood clots. Hence, the administration of anticoagulants coupled with thrombolytic drugs to impede the development and growth of blood clots is cardinal for patients.

In line with this, Indian scientists discovered bafibrinase, a serine proteinase of *Bacillus* sp., AS-S20-I with a molecular weight of 32.3 kDa, which exhibits thrombolytic and anticoagulant properties (Mukherjee et al., 2012). Thrombolytic activity along with plasminogen activation was shown by two proteolytic enzymes with molecular weights 29 and 29.5 kDa produced by *Bacillus halodurans* IND18 and *B. cereus* IND1, respectively (Vijayaraghavan and Vincent, 2014). The 20.5 kDa metalloprotease was purified from *B. subtilis* K42, which also showed fibrinolytic and anticoagulant potential (Hassanein et al., 2011). Subtilisin-like proteinase and glutamyl endopeptidase of *B. pumilus* 7P possess noticeable anticoagulant properties (Danilova et al., 2012).

With the development of post-genomic technologies, there has been a tendency to replace drugs of animal origin with affordable microbial drugs. In this regard, the intensive search for strains producing proteases with high fibrinolytic, thrombolytic, and anticoagulant activity is relevant. Thus, *Bacillus* proteinases are potential enzymes for use in the development of thrombolytic drugs.

### Microbial Enzymes Targeting Amyloid Plaques and Prion Complexes

Alzheimer’s disease (dementia) is one of the common pathologies of the central nervous system. Amyloid plaques containing amyloid beta protein, which also accumulates in the walls of the cerebral arteries, are found in the interstitial fluid of the brain. Amyloid plaques are deposits of beta-amyloid (Aβ) peptides, with a size of 42 amino acid residues (Hardy and Selkoe, 2002). Aβ is formed due to the enzymatic cleavage of its precursor (amyloid precursor protein, APP), usually present in the membranes of neurons and other cells.

A strain of the genus *Bacillus*, which produces a protease exhibiting high degradation activity against PrPSc, has been characterized. Sequence analysis showed that the serine protease of this strain belongs to the subtilisin family and has optimal pH and temperature ranges of 9–10 and 60–70°C, respectively. Western blot analysis showed that the protease is also able to decompose homogenate of brain affected with bovine spongiform encephalopathy. In addition, it was shown that protease much more effectively destroys dried PrPSc, firmly attached to a plastic surface, in comparison with proteinase K or the previously reported keratinase of *Bacillus licheniformis* PWD-1 (Yoshioka et al., 2007). Another study described a hyperthermostable protease, Tk-subtilisin, which successfully breaks down PrPSc under extreme conditions (Koga et al., 2014).

These data may be useful in studying the activity of bacillary proteinases, especially with respect to Aβ, which causes AD. For example, a Aβ peptide (42 amino acid residues) was cleaved by *B. pumilus* 7P proteinases (glutamyl endopeptidase, subtilisin-like proteinase, and metalloproteinase), resulting in the formation of a non-pathogenic form of the peptide that lacks the ability to form plaques. All studied proteinases hydrolyzed amyloid beta, but the most promising in practical application are glutamyl endopeptidase and metalloproteinase, which can inactivate the Glu11-His14 beta amyloid dimerization initiation site (Danilova et al., 2014).

The nattokinase of *B. subtilis natto* described earlier, proteinase K and Carlsberg subtilisin, have the ability to hydrolyze amyloid (Hsu et al., 2009). These proteins have also been offered as potential agents for the development of drugs against AD.

A multipurpose approach using biological preparations aimed at prion proteins opens up great prospects for the development of effective therapeutic interventions for AD.

### *Bacillus* Enzymes for Biofilm Destruction

It is now recognized that most microbes live in complex communities called biofilms. In a biofilm, individual cells are consolidated by an extracellular polymer matrix, usually consisting of polysaccharides, proteins, and DNA (Flemming et al., 2016). The biofilm lifestyle offers several benefits for microbes. For example, microorganisms within a biofilm can gain access to inaccessible nutrients and protect themselves against environmental fluctuations (Costerton et al., 1995). However, the negative repercussions in the formation of biofilms lies in the immense problem they present in health care, agriculture, and industry.

One of the methods to combat biofilms is the use of proteases. The anti-biofilm activity of bacillary proteases has already been tested on a large number of bacterial biofilms: *Pseudomonas*, *Acinetobacter*, and *Serratia* (Elchinger et al., 2015; Galie et al., 2018). For instance, a neutrase from *B. amyloliquefaciens* is known to exhibit endoprotease activity under neutral conditions (Elchinger et al., 2014). The protease from *B. licheniformis* (cited as Protease B) is a serine-type endoprotease that hydrolyzes most peptide bonds between pH 6.5 and 8.5, with an optimal temperature of 60°C, and is also used in biofilm regulation (Thallinger et al., 2013). The alkalase from *B. licheniformis* is a serine endopeptidase, mainly consisting of subtilisin A, which has been used in the paper industry to remove biofilms of various bacterial species (Marcato-Romain et al., 2012).

The effect of the proteolytic enzymes *B. pumilus* 3-19 on the structure of *Serratia marcescens* biofilms was studied. High efficacy of treatment with subtilisin-like protease and glutamyl endopeptidase for biofilm removal has been demonstrated. Enzymatic processing led to the degradation of EPS components and significant destruction of biofilms (Mitrofanova et al., 2017).

*Bacillus* spp. synthesize a large number of enzymes, amino acids, and other biologically active substances that destroy biofilms (Elchinger et al., 2015). The antibacterial activity of the antimicrobial peptide (CSpK14) isolated from *B. amyloliquefaciens* in combination with β-lactams against vancomycin-resistant strains of *S. aureus* (VRSA) and *Enterococci* (VRE) has been well established (Regmi et al., 2017). Chitosanase synthesized by *B. licheniformis* inhibited the biofilm formation of *Pseudomonas aeruginosa* and *Klebsiella pneumoniae* to about 22 and 29%, respectively, relative to a 100% growth in control samples. Thus, chitosanases have a promising advantage as an antibiotic agent, since they combat against biofilms formed by pathogenic bacteria (Muslim et al., 2016). An antimicrobial agent (protein BL-DZ1 with a molecular weight of 14 kDa) isolated from a tropical marine strain of *B. licheniformis* acts as a potential antibiofilm agent (Dusane et al., 2013).

Thus, *Bacillus* spp. are characterized by the wide range of antibiotic substances they produce, which in turn determine their high antagonistic activity against various microorganisms. Therefore, the creation of drugs based on bacilli or their metabolites is an important and promising aspect for destroying the integrity of biofilm matrices. Coupled with the use in medicine, it is important to obtain environmentally friendly products; therefore, new technologies based on bacilli are no less important in agriculture.

## Bacillary Enzymes in Agriculture

### As an Additive for Animal Feed

To increase the efficiency of animal feed use, microbial enzymes that increase the digestibility of nutrients are used (Choct, 2006). Commercially available feed enzymes include phytases, proteases, α-galactosidases, xylanases, and α-amylases, which are mainly used to supplement the feed of pigs and poultry (Selle and Ravindran, 2007). The logical candidates for generating feed enzyme products are non-pathogenic and non-toxigenic microbial strains (Pariza and Cook, 2010).

Microbial amylases and proteases are introduced into enzyme-based multi-enzyme complexes that enhance digestive function by complementing the action of similar endogenous gastrointestinal enzymes in birds. The sources of these enzymes are usually *B. amyloliquefaciens*, *B. licheniformis*, and *B. subtilis* (Contesini et al., 2018). *B. subtilis* is a cosmopolitan probiotic bacterium with an excellent enzymatic profile that can improve nutrient absorption and stimulate healthy growth. It has been described that *Bacillus* enzymes can help marine animals absorb nutrients from non-traditional and economical plant resources (Olmos Soto, 2017). It was also reported that using fermented soybean meal (SM) of *Bacillus pumillus* SE5 (BP; BPFSM) as a feed has a beneficial effect on antioxidant capacity, innate immunity, and intestinal health in young Japanese Sebastes (Rahimnejad et al., 2019).

Industrial poultry is the largest consumer of feed enzymes, which have been established as a tool that improves the efficiency of nutrient assimilation and energy (Romero et al., 2013).

Compounds such as phytates cause significant decrease in the nutritional value of animal feed. Phytases of bacteria of the genus *Bacillus* are promising as feed additives. These enzymes are characterized by elevated stability both thermally and in a wide range of pH (from 3.0 to 9.0); resistance to the action of proteolytic enzymes of the gastrointestinal tract; and high affinity for calcium ions, which are involved in the stabilization of the enzyme molecule and the formation of its active center (Balaban et al., 2016). This integration of properties makes bacillary phytases suitable for functioning in the digestive tract of birds (Lei et al., 2013). A β-propeller phytase of *Bacillus ginsengihumi* was previously isolated and characterized (Akhmetova et al., 2013). This *B. ginsengihumi* phytase was observed to thrive well in the acidic conditions similar to that the crop and small intestine in poultry (Dersjant-Li et al., 2015), as well as to have a thermal stability that corresponds to the conditions for the production of granular feed (65–80°С; Lei et al., 2013). The results of another study showed that adding subtilisin-like *B. pumilus* proteinase to the diet of broiler chickens has a positive effect on growth performance, nutrient digestibility, and meat quality (Koryagina et al., 2019).

An alternative to the outright delivery of these enzymes into the animal is also possible; bacteria that can synthesize extracellular enzymes directly in the digestive tract of the host organism are used as a feed additive. The nutritional effect of *B. subtilis* spores on growth rates, blood metabolites, antioxidant levels, and digestive enzyme activity in growing quail was studied (Latorre et al., 2016; Abdel-Moneim et al., 2019). It was found that the *B. subtilis* strain CM40 possesses the desired *in vitro* probiotic properties and may be a potential candidate for supplementation in animal feed (Mingmongkolchai and Panbangred, 2017). Three probiotic strains of *Bacillus* (*B. subtilis natto*, *B. licheniformis*, and *B. cereus*) showed favorable effects on chickens with minimal strain specificity (Gong et al., 2018). With recent bans on the growth-promoting use of antibiotics, alternative strategies are needed to improve the performance of agricultural animals (Brüssow, 2017). The reduced use of antibiotics in poultry has significantly increased the occurrence of subclinical necrotic enteritis (SNE) caused by *Clostridium perfringens* (CP), which compels researchers to seek alternatives to antibiotic growth stimulants (AGPs), in particular probiotics. It has been shown that the nutritional supplement based on *B. licheniformis* H2 can effectively prevent the occurrence of CPE-induced SNE and improve the growth rates of broiler chickens suffering from SNE, by improving intestinal development, antioxidant ability, and apoptosis (Zhao et al., 2019). It is also known that the genus *Bacillus* is widely used as probiotics in feed additives for pigs (*B. amyloliquefaciens* 15078, *Bacillus mojavensis* 10894, and *B. subtilis* 15130) and in aquaculture (*B. licheniformis* Dahb1, *B. pumilus*, and *Bacillus coagulans* B16; Jers et al., 2017; Kuebutornye et al., 2019). To obtain optimal effects, the correct choice of probiotic strains, as well as various combinations of enzymes, which are important for improving nutrition and protecting the health of domestic animals, is necessary. Further research in this direction is needed to expand the arsenal of drugs based on bacteria and their enzymes.

### Degradation of Feathers, Wool, and Hair by Bacillary Enzymes

Feathers of fowls have become one of the main pollutants due to their recalcitrant nature. A feather consisting of 90% keratin can be a good source of peptides, amino acids, and minerals for use as an organic fertilizer. The use of keratinolytic bacteria can become a sustainable and alternative tool for promoting and improving organic farming, agroecosystems, human health, soil biological activity, and the environment as a whole. Bacterial keratinases are serine-type proteases that exhibit optimal activity at a pH of 6–9 and a temperature range of 30–50°C (Tamreihao et al., 2019).

Effective feather decomposing bacteria were obtained and identified as *B. pumilus* GRK (Ramakrishna et al., 2017), *B. licheniformis* ALW1 (Abdel-Fattah et al., 2018), and *B. amyloliquefaciens* S13 (Hamiche et al., 2019). Proteases are of particular interest because of their effect on insoluble keratin substrates of various origins. Proteases are widely used in the leather industry to remove hair from animal skins. Excellent hair removal efficiency with proteinases from bacteria such as *B. mojavensis* SA has been reported (Hammami et al., 2018). Similar properties have likewise been established in *Bacillus aerius* NSMk2 (Bhari et al., 2019), *Bacillus thuringiensis* (Agasthya et al., 2013), *B. pumilus* SCU11 (Wang et al., 2016), *Bacillus* sp., SB12 (Briki et al., 2016), and *B. subtilis* KT004404 (Rehman et al., 2017).

## Summary

Microorganisms produce various types of extracellular enzymes for metabolic support, self-defense, and maintenance of normal physiological state. Microbial biotechnology, paralleled with the achievements of genetic and protein engineering, offer great opportunities for creating targeted enzyme-based biologically active substances. Proteolytic enzymes constitute one of the most diverse groups of microbial proteins in terms of properties. Thanks to the varied specificity and multiplicity of properties, these enzymes are widely used in biotechnology and medicine. The genus *Bacillus* is probably the most important bacterial source of proteases and is capable of producing high yields of neutral and alkaline proteolytic enzymes with remarkable properties, such as high resistance to extreme temperatures, pH, organic solvents, and oxidizing agents. At present, advancement in the use of new genomics and proteomics data to create new pharmaceutical drugs is widely observed. Much headway has been made in the development and implementation of technologies for the production of novel and safe feed additives that improve the health and productivity of agricultural animals, reduce the economic cost of feed, and improve the environment. Another important economic and environmental challenge is the use of feather as a raw material for the creation of effective feed and fertilizer additives. Several studies are aimed at solving these important practical problems.

## Author Contributions

All authors listed have made a substantial, direct, and intellectual contribution to the work and approved it for publication.

### Conflict of Interest

The authors declare that the research was conducted in the absence of any commercial or financial relationships that could be construed as a potential conflict of interest.
